# Real and simulated bioavailability of lead in contaminated and uncontaminated soils

**DOI:** 10.1186/2052-336X-12-108

**Published:** 2014-07-14

**Authors:** Marin Senila

**Affiliations:** 1 INCDO-INOE 2000, Research Institute for Analytical Instrumentation, ICIA, 67 Donath, 400293 Cluj-Napoca, Romania

**Keywords:** Lead, Bioavailability, Pollution, Soil-plant transfer, Diffusive Gradients in Thin-Films

## Abstract

**Background:**

Lead (Pb) is a toxic element that occurs in elevated concentrations in soils, mostly as a result of anthropogenic activities. This study assess the Pb bioavailability in soils from two areas with different contamination level using Diffusive Gradients in Thin-Films (DGT) technique, single extractions and metal contents of vegetables grown on contaminated soils.

**Results:**

In the area situated far from mining and smelting activities, the pseudo total Pb concentration (12 – 51 mg kg^−1^ dw) was found to be comparable to that normally found in unpolluted areas. In the area from the vicinity of the Pb smelter very high concentrations of pseudo-total Pb (850 – 9300 mg kg^−1^ dw) were found. The average concentrations of Pb accumulated in onion, garlic, carrot, and parsley grown on this contaminated soils were 18, 48, 38 and 91 mg kg^−1^ dw, respectively, and represent a risk factor for the consumers.

**Conclusions:**

The present study demonstrates the utility of DGT technique for the assessment of Pb bioavailability, since, generally, better correlations are obtained between the effective Pb concentration and Pb concentration in vegetables than for bioavailable Pb determined by chemical extractions and Pb concentration in vegetables.

## Background

Lead (Pb) pollution represents an important environmental and health issue all over the world, since it is very toxic and may causes disorders of nervous, reproductive and digestive systems [1,2]. Soils in the areas surrounding mining exploitations and ore processing facilities are affected by the increased content of toxic elements, which can enter in the food chain through plants uptake [3-5]. In north-western Romania, Pb was one of the most heavily exploited metals, causing elevated levels of Pb in soils from the vicinity of mining and ore processing sites [6].

Usually, environmental risk studies assess only the pseudo-total concentration of metals in soils and do not provide information related to bioavailable metal fractions. For the assessment of a metal’s bioavailability in soils, various single or sequential extraction schemes, based on different chemical extractants (e.g. neutral salt solutions, diluted acids, complexing agents) were developed and applied [7-10]. However, by applying these techniques, neither the decrease in metal concentrations at the root-soil interface nor the resupplying in the solid phase of the soil are taken into account [11]. The diffusive gradients in thin-films (DGT) [12] has the advantage to mimic more accurately the processes that take place at the soil-root interface, as the deployed DGT devices in soil decrease the local concentration of metals in the soil solution at the DGT-soil interface [13]. The DGT devices consist of a plastic assembly containing a layer of resin gel covered with a layer of diffusive gel through which labile species can freely diffuse, according to their specific diffusion coefficients. To calculate the concentration of bioavailable element in soil solution, a computer program, DGT-induced fluxes in sediments and soils (DIFS), has been developed [14]. This program is based on a numerical model which takes into account the DGT response to metal’s concentration in soil and to the resupplied metal from the soil solid phase to soil solution. By applying the DIFS, the effective concentration in soil (*C*_*E*_) can be calculated. Good correlations between metals effective concentration and their bioaccumulation in plants was reported even if the main limitation of DGT technique is that it does not take into account the root-induced processes in the rhizosphere which influence the metal’s mobility and bioavailability [15,16].

The aims of this study were: (1) to evaluate the Pb bioavailability in soils with different contamination levels in NW Romania using chemical extractions, DGT technique and vegetables Pb content and; (2) to evaluate the capacity of chemical extractions and DGT to accurately predict Pb bioavailability in contaminated and uncontaminated soils by studying the relationships between soil characteristics, real Pb bioavailability in soil assessed by bioaccumulation in plants and simulated Pb bioavailability assessed by DGT and chemical extractions.

## Methods

### Site description, soil and vegetable sampling

**Figure 1:**
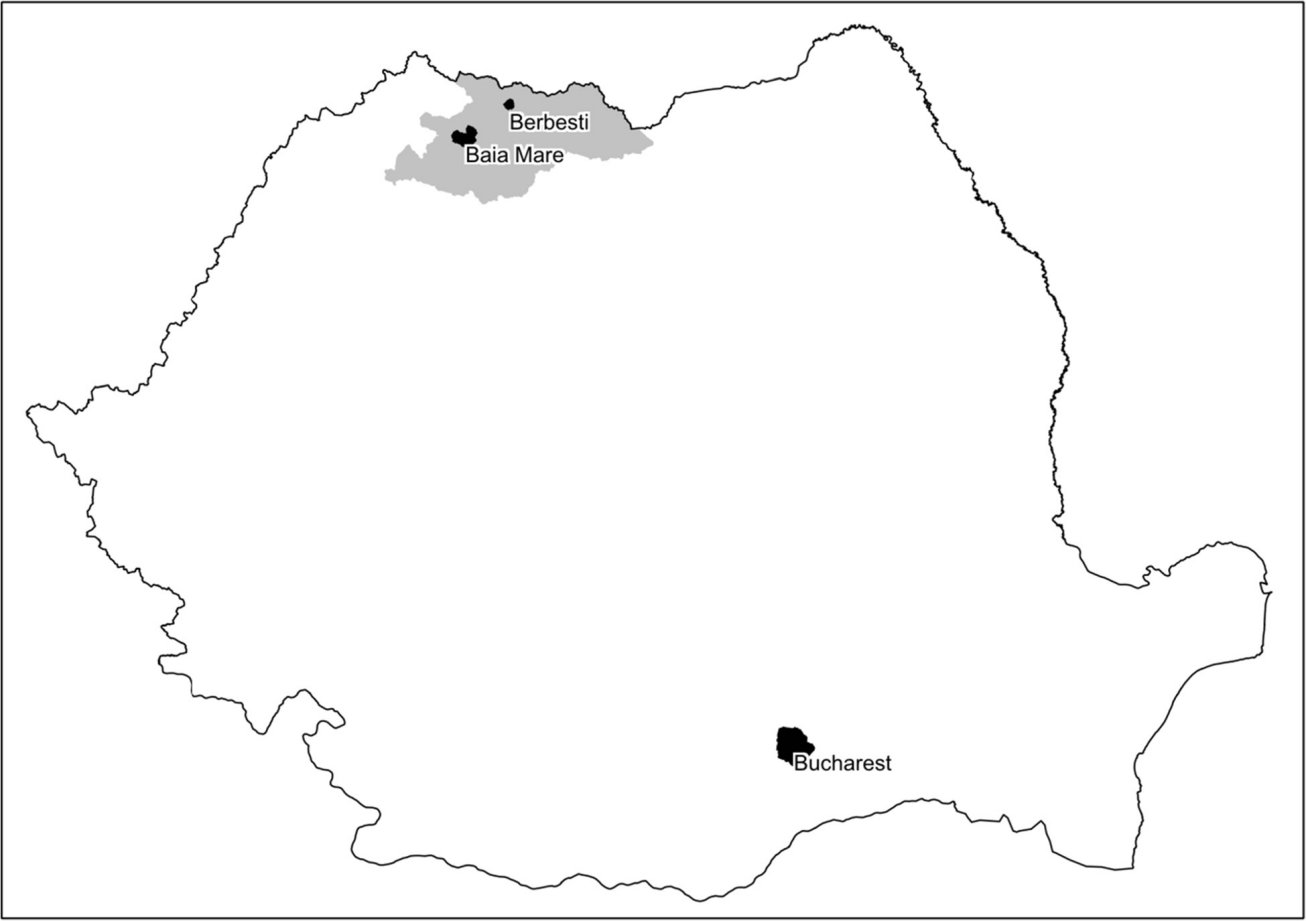
Study areas in Northwestern Romania (Baia Mare and Berbesti).

In summer 2011, soil and vegetables samples were taken from private gardens from two areas in NW Romania. Berbesti (BE) is a small village located between 23°49’- 23°51’E longitude and 47°50’- 47°52’N latitude is situated away from pollution sources. Ferneziu (FE), is a district in Baia Mare town, located between 23°53’- 23°63’ E longitude and 47°65’- 47°70’ N latitude, where, a Pb smelter functioned (Figure 1). The smelter generated metal rich wastes and dusts that in time build-up in surrounding soils. Even though currently the processing and smelting activities are considerably reduced, the surrounding soils are still contaminated with Pb. Several studies have reported the existence Pb contamination in this area [17-19] however limited information exists regarding the Pb bioavailability and the level of Pb accumulated in vegetables.

Roots and shoots of commonly grown vegetables: parsley (*Petroselinum hortense*), carrot (*Daucus carota L.*), onion (*Allium cepa L.*), and garlic (*Allium sativum L.*) were sampled in ten locations from each of the two study areas. Vegetable samples were washed with tap and distilled water. Soil underneath vegetables was also sampled and stored in polyethylene bags for transport to the laboratory. In the laboratory, the soils were air-dried, until constant weight, grounded and sieved through sieves with nylon meshes of 100 μm. The vegetables were dried at 40°C, and then ground to powder using a grinder.

### Reagents, standard solutions and CRMs

All reagents used were of analytical grade: 37% (w/w) HCl, 65% HNO_3_ (w/w), 30% H_2_O_2_ (w/w), NH_4_Cl salt (Merck, Germany). Calibration standards were prepared from the 1000 mg L^−1^ multielement IV ICP solution (Merck, Germany) by appropriate dilutions. DGT devices were purchased from DGT Research Ltd. (Lancaster, UK). Ultrapure water obtained using a Milli Q system (Millipore, France) was used for dilutions. A soil certified reference material (CRM) SRM 2709 San Joaquin Soil (National Institute of Standards and Technology, New York, USA) and a vegetable CRM NCS ZC 85006 Tomato (China National Analysis Center for Iron and Steel, Beijing, China) were used for the quality control of pseudo-total Pb determination. Percent recoveries (%) of Pb in soil and plant CRMs, calculated using the average of five replicates and the relative standard deviation were 95 ± 8% and 103 ± 15%, respectively. The relative standard deviation of reproducibility (s_R_), and the limit of reproducibility (R), calculated as 2.8 × s_R_ for pseudo-total Pb measurements ranged between 3.3 - 5.1% and 9.2 - 14.3%, respectively.

### Analytical methods

*Aqua regia* (HCl:NHO_3_ = 3:1 (v/v)) extraction was used for the determination of the pseudo-total metals (Pb, Fe, Mn, Al) concentrations of soils, according to ISO 11466:1995. An amount of 1 g of dried soil sample, was heated with 28 mL *aqua regia* for 2 hours, then filtered through 0.45 μm pore size filter, and diluted to 100 mL with ultrapure water. Single chemical extractions in diluted strong acid (1 M HCl) and neutral salt solution (1 M NH_4_Cl) were used for the estimation of potentially available Pb content from soil, according to the procedures previously described by Kashem et al. [20]. The vegetables Pb content was determined after digested by heating of 0.5 g dried sample with a mixture of 2 mL of H_2_O_2_ and 6 mL HNO_3_ in a MWS3+ Berghoff microwave oven (Eningen, Germany). The concentrations of metals in soil and vegetable extracts were measured by inductively coupled plasma optical emission spectrometer (ICP-OES) Optima 5300 DV (Perkin Elmer, USA) or by inductively coupled plasma mass spectrometer (ICP-MS) ELAN DRC II (Perkin Elmer, Canada). Soil pH was measured in 1:5 (w:v) soil to water ratio, using a 3340 ion-meter with a pH electrode (Jenway, UK). Total and inorganic carbon contents were measured using the Multi N/C 2100S Analyser (Analytic Jena, Germany), then the total organic carbon (TOC) was calculated as difference between total and inorganic carbon. Cation exchange capacity (CEC) was determined according to ISO 23470:2007, by measuring the extractable major cations using ICP-OES Optima 5300 DV (Perkin Elmer, USA). Water holding capacity (WHC) was determined gravimetrically, according to the method described by Muhammad et al. [11].

For DGT determinations, amounts of 25 g of soil samples were mixed with ultrapure water to 80-100% WHC, in plastic containers, and kept for 24 h at 22 ± 2°C, for equilibration. The DGT devices were introduced into the soil slurries and kept for 24 h at 22°C. After retrieval, the DGTs were carefully washed with ultrapure water and the resin gels were introduced in 15 mL centrifuge tubes for metal elution in 1 mL of 1 M HNO_3_ for minimum of 24 h. The eluents were quantitatively diluted 5 times before the determination of the metal concentrations by ICP-MS. The mass (M) of Pb accumulated in the resin, was calculated using the Eq. 1 [13]:

(1) $$M=C\times \frac{V_{\mathrm{eluent}}+V_{\mathrm{gel}}}{f_{e}}$$

where *C* is the metal concentration in 1 M HNO_3_ (in μg L^−1^), as determined by ICP-MS, *V*_*eluent*_ is the volume of diluted eluent (5 mL), *V*_*gel*_ is the volume of resin gel (0.15 mL), *f*_*e*_ is the elution factor (0.8). The average time concentration of metal (*C*_*DGT*_) was calculated according to Eq. 2:

(2) $$C_{\mathrm{DGT}}=\frac{M\times \Delta g}{D\times t\times A}$$

where *M* is the mass of metal from Eq. 1, *Δg* is the thickness of the diffusive gel + membrane filter, *D* is the diffusion coefficient of the Pb in the resin gel at 22°C (7.40 E^−6^ cm^2^ sec^−1^), *t* is the deployment time in seconds, and *A* is the area of the sampling window of the DGT device (3.14 cm^2^, in our case).

The effective concentration, (*C*_*E*_) was calculated using Eq. 3:

(3) $$C_{E}=\frac{C_{\mathrm{DGT}}}{R_{\mathrm{diff}}}$$

where *C*_*DGT*_ was calculated using Eq. 2, while *R*_*diff*_ was calculated using the numerical model DIFS and represents a measure of the depletion degree of the concentration at the interface of DGT device and soil for the diffusion-only-case. The input parameters to calculate *R*_*diff*_ were the coefficients of metal diffusion in water, in soil and in diffusive gels (*D*_*0*_, *D*_*s*_, *D*_*d*_), diffusion layer thickness (*Δg*), deployment time (*t*), particle concentration (*P*_*c*_) and the typical value of particle density (*P*_*s*_) for mineral soils, of 2.65 g cm^−3^[21]. A large value for *T*_*c*_ (soil response time), of 1 × 10^9^ and a small value for *K*_*d*_ (plant available fraction of element bound to the soil) of 1 × 10^−9^ were introduced in the input mode of 2D DIFS in order to calculate *R*_*diff*_.

To measure the metal concentration in soil solution (*C*_*soln*_), portions of the soil slurries prepared for the DGT measurements, were introduced in polyethylene tubes and centrifuged at 5000 rpm for 20 minutes, using a Universal 320 centrifuge (Hettich, Germany). The supernatants were filtered through 0.45-μm pore size filters and acidified with 10 μL ultrapure 65% HNO_3_ prior to ICP-MS determination.

### Statistical analysis

The XLStat Microsoft Excel plug-in (Addinsoft) was used for the statistical processing of the data. Linear regression analysis was carried out to find correlations between soil properties, potentially available Pb, *C*_*E*_ and Pb accumulated in shoots or roots of vegetables.

## Results and discussion

### Chemical characteristics of soils

The soil chemical characteristics (pH, TOC, CEC and pseudo-total extractable Pb, Al, Mn and Fe) are presented in Table 1. The average soil pH was found to be neutral in both areas (6.9 in FE and 7.3 in BE, respectively), except for one sample in the vicinity of the Pb smelter, where the soil was slightly acidic (5.5). The CECs ranged between 17.7 – 30.3 cmol kg^−1^ in BE, and 14.7 - 27.4 in FE, while the TOC was in the range of 3.8 – 8.7% dw in BE and 2.4 – 7.0% dw in FE. Positive correlations were found between CEC and TOC, both in contaminated and uncontaminated areas, which is usually observed in soils [10]. Due to the anthropogenic input of Pb by atmospheric deposition, very high concentrations of pseudo-total Pb were found in the soils near the Pb smelter (FE), with an average value 32 times higher than the intervention threshold (100 mg kg^−1^ dw), established by Romanian legislation for soils in residential and agricultural areas [22]. The average pseudo-total Pb concentration in FE area was comparable with those reported by Bosso and Enzweiler [23] in soils from a mining district in Brazil, by Romero et al. [24] in soils from the vicinity of a smelter from Mexico and by Anjos et al. [10] in soils from the Bracal lead mine area, Portugal. However, our results were higher than those from soils around a Pb-Zn mine in Spain [25]. The pseudo-total Pb in soils from BE area situated far away from the pollution sources, ranged between 12 – 51 mg kg^−1^ dw and in all samples, was below the intervention threshold established by the Romanian legislation. Moreover our values were of the same order of magnitude as the average value of 15 mg kg^−1^ dw, considered as naturally arising Pb in the Earth’s crust [26]. The mean concentrations of Al, Fe and Mn were of about two times higher in FE than mean concentrations found in BE. Spatial distribution of Pb and Mn were positive correlated, suggesting that Pb has a preferential association with minerals containing Mn.

**Table 1:** General characteristics of garden soils collected from the study area

**Sites**		**pH**	**TOC (%)**	**CEC (cmol kg**^**−1**^**)**	**Pseudo-Total concentration (mg kg**^**−1**^**dw)**			
					**Pb**	**Al**	**Mn**	**Fe**
FE	Mean	6.9	5.1	22.6	3170	30000	1230	32600
	Range	5.5-7.7	2.4-7.0	14.7-27.4	850-9300	21300-35400	872-1600	26100-38000
BE	Mean	7.3	5.5	22.7	29.4	17120	727	19200
	Range	6.8-7.8	3.8-8.7	17.7-30.3	12.0-51.0	12400-21200	494-897	13200-23500
AL*		-	-	-	50	-	-	-
IL**		-	-	-	100	-	-	-

*AL-alert level for soil in sensitive (residential and agricultural) areas (MO 856:1997).

**IL- intervention level for soil in sensitive (residential and agricultural) areas.

### Pb extractability in diluted strong acid and in neutral salt solution

**Figure 2:**
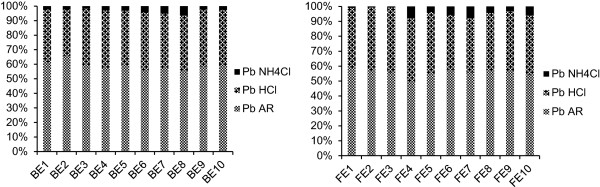
Pb extraction in aqua regia, 1 M HCl and NH4Cl solution in soil from BE and FE.

As shown in Figure 2, the average percentages of Pb extractabilities in 1 M HCl were of about 70%, similar to those reported by Kashem et al. [20]. Strong linear positive correlations were observed between the pseudo-total content of Pb in soil and the Pb extracted by diluted HCl, both for the polluted and non-polluted areas. The strong correlations between *aqua regia* and HCl extractable Pb can be explained by the fact that both 1 M HCl and *aqua regia* mixture can attack the potentially mobile Pb from soil, but not the Pb bounded in the silicate matrix.

In the polluted area, the 1 M NH_4_Cl extractible Pb concentrations, were in the range of 12.1 - 918 mg kg^−1^ dw (average concentration of 276 mg kg^−1^ dw), with an average percentage of Pb extractability of about 8.7%, while in the unpolluted area, the averages of Pb extractability in 1 M NH_4_Cl was about 5.0%.

The stronger correlation between the neutral salt soluble and pseudo-total Pb in the polluted area content, than in non-polluted area could be explained by the more recent origin of the Pb. Pb forms strong complexes with dissolved organic matter by COO^−^ groups that are capable to maintain Pb in soil solution. In time, the soluble organic compounds are transformed into compounds with higher molecular weight [27], able to retain Pb by chelation, thus reducing Pb mobility.

### Pb bioavailability assessed by DGT technique

Pb concentrations measured by DGT technique (*C*_*DGT*_), Pb in soil solution and calculated effective concentrations are presented in Table 2. The *R* ratio between *C*_*DGT*_*/C*_*soln*_ indicates the capacity of the solid phase of soil to re-supply metals in the pore water. The values of *R* ratio range from 0 to 1, and a high value of this parameter (*R* > 0.95) indicates that the capacity of the solid phase to resupply the pore water is high, since a low R value shows a very limited metal resupply from the solid phase [13,28]. The *R* ratios for Pb in the polluted area (FE) ranged between 0.58 - 0.90 (average 0.75), while in the unpolluted area (BE) ranged between 0.20 – 0.86 (average 0.59), indicating in general a higher re-supply of Pb from solid phase in polluted soils.

**Table 2:** **Pb contents extracted in 1 M HCl, 1 M NH**_**4**_**Cl, soil solutions (C**_**soln**_**), Pb measured using DGT technique (C**_**DGT**_**) and effective concentration of Pb (*C***_***E***_**) calculated using DIFS (Mean ± SD, n = 3)**

**Sites**	**C**_**1M HCl**_	**C**_**1M NH4Cl**_	**C**_**soln**_	**C**_**DGT**_	**CE**
	**(mg kg**^**−1**^**dw)**	**(mg kg**^**−1**^**dw)**	**(μg L**^**−1**^**)**	**(μg L**^**−1**^**)**	**(μg L**^**−1**^**)**
FE1	2250 ± 190	59.4 ± 3.62	105 ± 9.22	63.0 ± 6.60	2020 ± 216
FE2	940 ± 95.2	13.2 ± 0.93	188 ± 12.5	156 ± 18.8	4940 ± 629
FE3	660 ± 46.6	12.1 ± 1.04	49.1 ± 3.09	38.1 ± 4.99	1240 ± 165
FE4	1560 ± 120	287 ± 21.1	208 ± 12.1	146 ± 16.4	4830 ± 556
FE5	3620 ± 266	324 ± 25.5	332 ± 25.5	202 ± 20.8	6620 ± 689
FE6	5950 ± 365	918 ± 55.6	245 ± 21.7	221 ± 23.3	7110 ± 774
FE7	3500 ± 321	752 ± 53.2	55.9 ± 5.17	32.6 ± 4.75	1050 ± 161
FE8	1410 ± 110	161 ± 12.2	103 ± 9.13	88.7 ± 10.9	2880 ± 356
FE9	1260 ± 125	100 ± 10.8	144 ± 12.8	112 ± 10.6	3650 ± 342
FE10	874 ± 77.2	132 ± 12.8	186 ± 15.3	145 ± 15.7	4750 ± 717
BE1	16.0 ± 2.33	1.00 ± 0.12	1.58 ± 0.09	1.02 ± 0.12	33.4 ± 4.01
BE2	24.8 ± 3.12	1.84 ± 0.23	4.62 ± 0.24	3.63 ± 0.36	121 ± 11.3
BE3	14.0 ± 2.06	0.60 ± 0.05	2.34 ± 0.19	2.01 ± 0.28	66.6 ± 8.99
BE4	20.3 ± 2.11	1.61 ± 0.18	4.81 ± 0.33	1.61 ± 0.18	54.6 ± 6.03
BE5	14.8 ± 1.56	1.24 ± 0.16	2.97 ± 0.21	0.58 ± 0.08	19.2 ± 2.66
BE6	11.3 ± 1.23	1.28 ± 0.12	3.35 ± 0.31	1.99 ± 0.25	66.1 ± 8.04
BE7	13.4 ± 1.26	1.84 ± 0.19	2.56 ± 0.21	1.18 ± 0.13	40.0 ± 4.21
BE8	8.20 ± 1.11	1.40 ± 0.17	1.92 ± 0.13	1.05 ± 0.13	34.3 ± 4.09
BE9	29.6 ± 3.02	1.82 ± 0.20	4.55 ± 0.34	3.33 ± 0.49	107 ± 16.0
BE10	32.0 ± 2.83	2.20 ± 0.25	6.13 ± 0.51	4.25 ± 0.48	194 ± 15.7

The values for the particle concentration (*P*_*c*_) used for *R*_*diff*_ calculation ranged between 3.63 – 4.32 g cm^−3^ in soils from FE, while in the soils from BE, *Pc* values were within the range of 3.95 – 4.81 g cm^−3^. Using DIFS numerical model, the values of *R*_*diff*_ calculated for soils from FE ranged between 0.0302 – 0.0316 and between 0.0219 – 0.0310 in BE. The effective concentrations (*C*_*E*_) of Pb in the unpolluted area ranged between 19.2 – 194 μg L^−1^ (average 37.3 μg L^−1^) in BE, being in the same order of magnitude with those reported by Soriano-Disla et al. [29] for Pb in uncontaminated soils. In the polluted soil from FE, *C*_*E*_ values ranged between 1050 – 7110 μg L^−1^ (average 3910 μg L^−1^), being about 100 times higher than in unpolluted area. The low bioavailability of Pb in soils shows that this element is found as poorly soluble phases.

### Pb accumulation in roots and shoots of vegetables

The Pb concentrations measured in roots and shoots of the four analyzed vegetable species, expressed as mg kg^−1^ dw, are presented in Table 3. In the unpolluted area, the Pb concentrations, both in shoots and roots, were much lower than the value of 30 mg kg^−1^ considered toxic for plants [26]. In order to compare the concentrations of Pb in the edible parts of analyzed vegetables with the maximum level of allowable lead of 0.10 mg kg^−1^ fresh weight (fw) in vegetables (excluding leaf vegetables, fresh herbs, for which the maximum level of Pb is 0.30 mg kg^−1^ fw), established by the European Commission (EC 1881/2006), it was calculated the Pb concentration in vegetables as mg kg^−1^ fw taking into account the moisture content. Pb contents in onion shoots in the uncontaminated area were generally below the maximum limit of 0.10 mg kg^−1^ fw. In the majority (90%) of analyzed garlic roots, Pb concentration was above the maximum limit. Also, in carrot roots, the maximum allowable limit was exceeded in 50% of samples. In parsley shoots the maximum limit of 0.30 mg kg^−1^ fw was not exceeded. Pb concentrations in vegetables grown on the soils from the polluted area were frequently above the value considered toxic for plants (30 mg kg^−1^). Our results were in the same order of magnitude with average value (80.1 mg kg^−1^ dw) reported by Kisku et.al [30] for vegetables grown on soils from an industrial area from India, and those reported by Gupta et al. [31], in vegetables grown on soils irrigated with wastewater in India, but higher than the values reported by Khan et al. [32], for vegetables in China (2.55 – 4.50 mg kg^−1^ dw). Higher concentrations (409 mg Pb kg^−1^ dw) were reported by Türkdogan et al. [33] in vegetables grown on contaminated soils in Turkey.

**Table 3:** **Pb concentrations in roots and shoots of vegetables (mg kg**^**−1**^**dw) (Mean ± SD, n = 3)**

**Sites**		**Onion**		**Garlic**		**Carrot**		**Parlsley**	
		**Roots**	**Shoots**	**Roots**	**Shoots**	**Roots**	**Shoots**	**Roots**	**Shoots**
FE	Mean	18	8.3	48	16	38	16	91	26
	Range	5.2-46	1.8-23	4.7-120	2.5-29	9.8-79	3.6-34	18-340	8.7-42
BE	Mean	0.27	0.14	0.38	0.13	0.37	0.13	0.81	0.31
	Range	0.11-0.79	0.05-0.31	0.11-0.65	0.04-0.31	0.09-0.97	0.04-0.26	0.22-1.88	0.13-0.44

### Correlations between Pb concentration and bioavailability in soil and accumulation by plants

Roots are in direct contact with soil and it is expected to be better indicators of metals bioavailability in soils than the shoots, for which the metal accumulation is affected by the translocation processes, generally controlled by plant physiology [34]. As presented in Table 4, in the unpolluted area, the Pb accumulated in vegetables was generally better correlated with Pb extracted by different procedures than in the polluted area. The significant positive correlations suggest that the single extractions are the most suitable for the evaluation of Pb accumulation in vegetables. As presented in Table 5, in the polluted area no significant correlations between Pb content in vegetables and extraction methods were found. This is probably due to the high variability of the pseudo-total content and availability of Pb in soil samples in the polluted area, but also due to the physiological response of the plants grown on extremely contaminated soils, that can act as a barrier for the absorption of Pb, while the chemical extractants or DGT devices ignore this physiological response.

**Table 4:** Pearson correlation matrix between Pb extracted by chemical extractions, DGT, soil properties and pseudo-total Pb accumulation in the studied vegetable in uncontaminated area, Berbesti

**Variables**	**Pb AR**	**Pb HCl**	**Pb NH4Cl**	**Pb Csoln**	**Pb CE**	**pH**	**TOC**	**CEC**
Pb AR	1							
Pb HCl	0.96***	1						
Pb NH4Cl	0.64*	0.66*	1					
Pb Csoln	0.80**	0.85**	0.75*	1				
Pb CE	0.83**	0.84**	0.63*	0.83**	1			
pH	−0.61	−0.61	−0.37	−0.71*	−0.82**	1		
TOC	0.64*	0.69*	0.21	0.39	0.69*	−0.53	1	
CEC	0.79**	0.71*	0.25	0.52	0.71*	−0.60	0.85**	1
Pb garlic r	0.22	0.21	0.27	0.45	0.61	−0.80**	0.20	0.20
Pb garlic s	0.73*	0.72	0.65*	0.70*	0.85**	−0.82**	0.58	0.55
Pb onion r	0.61	0.63*	0.54	0.74*	0.87**	−0.80**	0.66*	0.72*
Pb onion s	0.80**	0.67*	0.50	0.67*	0.84**	−0.80**	0.49	0.74*
Pb parsley r	0.75*	0.78**	0.64*	0.74*	0.94***	−0.72**	0.63*	0.61
Pb parsley s	−0.36	−0.36	0.01	−0.18	0.03	−0.08	−0.07	−0.17
Pb carrot r	0.43	0.54	0.35	0.53	0.58	−0.43	0.32	0.20
Pb carrot s	0.29	0.41	−0.16	0.32	0.43	−0.32	0.69*	0.62

*, ** and *** - significant correlation at levels p < 0.05, p < 0.01 and p < 0.001, respectively.

*r* and *s* - roots and shoots, respectively.

**Table 5:** Pearson correlation matrix between Pb extracted by chemical extractions, DGT, soil properties and pseudo-total Pb accumulation in each studied vegetable in contaminated area, Ferneziu

**Variables**	**Pb AR**	**Pb HCl**	**Pb NH**_**4**_**Cl**	**Pb C**_**soln**_	**Pb C**_**E**_	**pH**	**TOC**	**CEC**
Pb AR	1							
Pb HCl	0.99***	1						
Pb NH_4_Cl	0.91***	0.89***	1					
Pb C_soln_	0.35	0.42	0.21	1				
Pb C_E_	0.40	0.44	0.29	0.95***	1			
pH	−0.37	−0.40	−0.51	−0.31	−0.35	1		
TOC	0.53	0.48	0.35	0.01	0.12	0.26	1	
CEC	0.30	0.27	0.13	0.12	0.14	0.41	0.80**	1
Pb garlic r	0.11	0.09	−0.06	−0.33	−0.32	−0.06	−0.19	−0.41
Pb garlic s	0.36	0.30	0.22	−0.28	−0.16	0.04	0.41	0.20
Pb onion r	−0.23	−0.21	−0.45	−0.03	−0.10	0.10	−0.20	0.00
Pb onion s	−0.13	−0.09	−0.38	−0.02	−0.15	−0.01	−0.31	−0.23
Pb parsley r	0.00	−0.01	−0.27	−0.20	−0.24	0.11	0.02	0.11
Pb parsley s	−0.08	−0.12	−0.11	0.07	0.29	−0.01	0.08	−0.03
Pb carrot r	0.37	0.34	0.32	0.17	0.31	−0.34	−0.09	−0.16
Pb carrot s	−0.37	−0.41	−0.49	−0.33	−0.29	−0.31	−0.14	0.01

** and *** - significant correlation at levels p < 0.01 and p < 0.001, respectively.

*r* and *s* - mean roots and shoots, respectively.

Overall, in our study, the effective concentrations measured by trapping available Pb from soil using DGT devices provides the best correlation coefficients between accumulation in vegetables and bioavailable fraction from uncontaminated soil. Our results are similar to those reported by Soriano-Disla et al. [29] who reported good correlation between effective concentrations of Pb and its accumulation in plants. However, in highly contaminated soils, *C*_*E*_ did not provide good predictions for the bioaccumulation of Pb. Similar behaviours were reported by Almas et al. [35] for Zn and Cd and by Tandy et al. [36] for Cu and Zn, who showed that DGT is not a good indicator for plants uptake in soils with high concentrations of metals.

## Conclusions

This study represents one of the first applications of the DGT technique for the assessment of Pb bioavailability in soil, in field conditions. Two areas with different exposure to the Pb pollution were studied. In the area situated far from mining and smelting activities, the level of pseudo-total Pb was similar to that commonly found in soils, while in soils from the vicinity of a Pb smelter, very high concentrations of pseudo-total Pb were found, which conducted to high amounts of bioavailable Pb in soils and high quantities of Pb accumulated in the edible parts of the vegetables, which represent a risk for the consumers. The present study demonstrates the utility of DGT technique for the assessment of Pb bioavailability, since generally, between effective concentration and accumulation in vegetables better correlations were obtained than between the available Pb estimated by chemical extractions and Pb content of vegetables. However, for the highly contaminated soils no significant correlations were found between bioaccumulation and Pb assessed by chemical extractions, even if DGT technique was used. Thus, further research is required to study the effectiveness of DGT, or other chemical assays, for the accurate prediction of Pb bioavailability in highly polluted soils.

## Competing interests

The authors declare that they have no competing interests.

## Authors’ contributions

MS organized the experimental setting, collected the samples, performed metals determinations, DGT and chemical extractions, interpreted the results and wrote the manuscript.
